# A “Talking” between Gold Nanoparticle and a Luminescent Iridium(III) Complex: A Study of the Effect Due to the Interaction between Plasmon Resonance and a Fluorophore

**DOI:** 10.3390/nano14191543

**Published:** 2024-09-24

**Authors:** Angela Candreva, Loredana Ricciardi, Elisabeta I. Szerb, Massimo La Deda

**Affiliations:** 1 Department of Chemistry and Chemical Technologies, University of Calabria, I-87036 Rende, Italy; angela.candreva@unical.it; 2 CNR-NANOTEC Institute of Nanotechnology, National Research Council, I-87036 Rende, Italy; loredana.ricciardi@cnr.it; 3 Coriolan Dragulescu Institute of Chemistry, Romanian Academy, 24, Mihai Viteazu Bvd., 300223 Timisoara, Romania; eszerb@acad-icht.tm.edu.ro

**Keywords:** nanomaterials, plasmon resonance, fluorophore, iridium compound

## Abstract

This paper explores a novel synthesis and characterization of silica-coated gold nanorods (AuNRs) embedding a highly emissive cyclometalated iridium(III) complex, denoted as Ir_1_. We investigate the optical properties and the interplay between the metal compound and gold plasmon, observing how the emission of Ir_1_ incorporated into the nanoparticles shows two emission bands, one in the blue and the other in the green-orange range of the visible spectrum. To obtain a clearer picture of what we were observing, we synthesized analogous nanosystems, from which it was possible to highlight the effect of different features. Based on what we observed, we proposed that the fraction of the iridium(III) complex in direct contact with the surface of the gold nanoparticle undergoes a “demixing” of the excited state, which, for cyclometalated iridium complexes, is generally considered a mixed LC+MLCT state. This preliminary study sheds light on the complexity of the “talking” between a fluorophore and a plasmonic system, highlighting the importance of considering the emitter typology when modeling such systems.

## 1. Introduction

Metal nanoparticles (MNPs) have attracted considerable attention for their ability to interact with light from the visible to the near-infrared (NIR) through the generation of resonant surface plasmons [1,2,3]. In recent years, many efforts have been made to understand and exploit plasmonic properties and their interaction with other classes of materials, as well as their functional applications [4]. The interaction between metal nanoparticles and luminescent molecules has received particular attention, and this combination has led to various applications in different fields, from photovoltaics to biomedical [5,6].

Interacting with a fluorophore, MNPs can significantly influence the molecule properties, and, vice versa, the latter can influence the plasmon resonance. This “talking” is mediated by the surface electron oscillations of the MNPs coupled with the electronic transitions of the light-emitting molecules. Indeed, the proximity of a metal nano-core alters the excited deactivation pathways of the surface-bound molecules. One of the remarkable properties of luminescent molecules when bound to metal surfaces is the reduced excited-state lifetime as a result of energy transfer from excited dye molecules to bulk metal, and, depending on the distance between the fluorophore and the metal core, the emission intensity can be increased or decreased. Several models have been proposed to describe these interactions [7]; in general, fluorescence resonance energy transfer (FRET) is used to explain the emission quenching [8,9], or metal enhanced fluorescence (MEF) is used to model emission enhancement [10,11,12]; however, in neither case is the specific identity of the fluorophore taken into consideration.

From an experimental point of view, it becomes essential to have synthetic methods that allow one to organize chromophores with specific properties and functions on MNPs so as to produce photoresponsive organic–inorganic nano-hybrid materials. Careful synthetic design allows one to functionalize the surface of the nanoparticles with luminescent molecules, ensuring a close contact between them, or to place the fluorophore at a well-defined distance from the metal surface, using spacers such as silica. In this way, the “talking” between the plasmon and the luminophore (FRET, MEF, etc.) can be controlled.

Among MNPs, gold nanoparticles (AuNPs) have received much attention, due to their characteristic plasmon resonance in the Vis–NIR range and to their versatile procedures for obtaining homogeneous nanoparticles such as, among others [13], microemulsion [14] and seed-mediated growth methods [15,16]

Nanoparticles can be coated with various coating agents depending on the environment in which they are to be dispersed [17,18]. Additionally, coating agents can act as functionalizers, giving the nanoparticles new properties and enabling their application in fluorescence imaging, biosensing and phototherapy [19,20,21,22,23]. Silica coating represents a powerful strategy for functionalizing gold nanoparticles [24,25,26]; by encapsulating the AuNPs within a silica shell, their stability is significantly enhanced. Furthermore, the silica shell is a useful platform for further functionalization with biomolecules, polymers, or obtaining ligands [27,28]. This opens up opportunities for applications in obtained drug delivery, where the AuNPs can be engineered to selectively bind to diseased cells or tissues, delivering therapeutic payloads with precision and efficacy [29,30,31]. Moreover, the silica shell can serve as a host for embedding luminescent molecules, creating hybrid nanostructures with synergistic optical properties [32,33].

We have previously reported the synthesis and characterization of gold-core silica-shell nanospheres embedding, within the polysiloxane matrix, a highly luminescent and photosensitizing iridium(III) complex, indicated briefly as **Ir_1_** (whose molecular structure is reported in Figure S1), to obtain an all-in-one nanoplatform for simultaneous imaging and phototherapy [34,35,36,37]. In general, iridium(III) complexes have been extensively studied for their rich and sensitive photophysical properties, mainly characterized by high phosphorescence quantum yields and sensitizing abilities [38,39,40].

On this basis, the synthesis and characterization of gold-core silica-shell nanorods encapsulating a photophysically-active **Ir_1_** is proposed herein. To favor the solubility of the nanosystems in aqueous environments, silica-coated AuNPs were overcoated by 3-(triethoxysilyl)propylsuccinic anhydride (TPSA), which makes nanoparticles very stable in water. Compared with spheres, nanorods possess a tunable aspect ratio, providing enhanced absorption in the NIR region, where tissue penetration is maximized, promoting the use of such nanosystems in the phototherapeutic field [41]. Interestingly, the photophysical properties of the newly synthesized gold-core silica-shell nanorods, AuNR@(**Ir_1_**)SiO_2_@TPSA, appeared unusual, probably due to the peculiarity of the fluorophore—a cyclometalated Ir(III) compound—and to the architecture of the nanosystem. To obtain a more detailed picture, similar nanosystems were prepared, in order to isolate the contributions of the various components; in particular, and with a different synthesis method, an analogous system with a spherical gold core (AuNS@(**Ir_1_**)SiO_2_@TPSA) was prepared. To highlight the role of the gold core, silica nanoparticles (without the gold core) incorporating **Ir_1_** was synthesized ((**Ir_1_**)SiO_2_@TPSA), while a nanosystem with a PEG-SH coating was prepared to highlight the role of the coating on the position of the plasmon band (AuNR@PEG-SH). By comparing the emission spectra and the lifetimes of the iridium(III) complex in the different nanosystems, a new effect caused by the interaction between the plasmon resonance of the nanoparticles and the luminescence of the fluorophore was observed and described.

## 2. Materials and Methods

Triton X-100, n-hexanol, cyclohexane, sodium 2-mercaptoethanesulfonate (MES), hexadecyltrimethylammonium bromide (CTAB, ≥96%), 5-bromosalicylic acid (5-BrSA, technical grade, 90%), O-[2-(3-Mercaptopropionylamino)ethyl]-O′-methylpolyethylene glycol (PEG-SH), hydrogen tetrachloroaurate trihydrate (HAuCl_4_·H_2_O, ≥99.9%), silver nitrate (AgNO_3_, ≥99.0%), L-ascorbic acid (≥99%), sodium iodide (99.99%), trisodium citrate (99%), sodium borohydride (NaBH_4_, 99%), isopropanol, tetraethoxysilane (TEOS), (3-aminopropyl)triethoxysilane (APTES), NaOH (98%), and MeOH were purchased from Aldrich, St. Louis, MO, USA. NH_4_OH (25% in water) was purchased from Chimie Plus, Saint-Paul-de-Varax, France. N-(3-triethoxysilyl)propylsuccinic anhydride (TPSA, 94%) was purchased from ABCR, Karlsruhe, Germany. Only homemade Milli-Q water, (ρ > 18 MΩ) was used for the preparation of the aqueous solution.

All of the chemicals were used as received. All glassware was washed with aqua regia, rinsed with water, sonicated 3-fold for 3 min with Milli-Q water, and dried before use. Iridium(III) complex was synthesized according to the procedure previously reported [42].

The absorption spectra have been obtained using a Perkin Elmer Lambda 900 spectrophotometer (Shelton, CT, USA).

Steady-state emission spectra were registered by a HORIBA Jobin-Yvon Fluorolog-3 FL3-211 spectrometer (Edison, NJ, USA), equipped with a 450 W Xenon arc lamp, double-grating excitation and single-grating emission monochromators (2.1 nm/mm dispersion; 1200 grooves/mm), and a Hamamatsu R928 photomultiplier tube (Hamamatsu City, Japan). Emission and excitation spectra were corrected for source intensity (lamp and grating) and emission spectral response (detector and grating) by standard correction curves.

Time-resolved measurements were carried out using the time-correlated single-photon counting (TCSPC) option on the Fluorolog 3. A nanoLED at 379 nm, fwhm < 200 ps with repetition rate at 1 MHz, was employed to excite the sample. Excitation source was directly fixed on the sample chamber at 90° to a single-grating emission monochromator (2.1 nm/mm dispersion; 1200 grooves/mm) and the emission was recorded with a TBX-04-D single-photon-counting detector. The photons collected at the detector were correlated by a time-to-amplitude converter (TAC) to the excitation pulse. Signals were recorded using an IBH Data Station Hub photon counting module, and data analysis was performed using the commercially available DAS6 software (version 6.4, HORIBA Jobin Yvon, Edison, NJ, USA). The quality of the fit was assessed by minimizing the reduced χ^2^ function and by visual inspection of the weighted residuals.

A transmission electron microscope (Jeol JEM-1400 Plus 120 kV, Tokyo, Japan) was used to measure the size and morphology of the gold nanoparticles. The samples for transmission electron microscopy (TEM) were prepared by the deposition of a drop of diluted solution on 300 mesh copper grids. The particles were observed at an operating voltage of 80 kV after evaporation of the solvent in air.

All experiments were conducted at 30 °C. 

## 3. Results and Discussion

**Synthesis of AuNR@CTAB**

Following the seed-mediated growth synthetic procedure, gold nanorods with an aspect ratio of 4 (48 nm × 12 nm, long axis × short axis, respectively) were synthesized [43,44].

*Preparation of the seed solution.* An amount of 25 μL of 5.0 × 10^−2^ M HAuCl_4_ water solution was mixed with 4.7 mL of 0.1 M CTAB water solution. An amount of 300 μL of a freshly prepared 1.0 × 10^−2^ M NaBH_4_ water solution was then added under intense stirring.

*Preparation of the growth solution*. An amount of 45 mg of 5-BrSA was added to 50 mL of 0.05 × 10^−2^ M CTAB water solution. When completely dissolved, 480 μL of 1.0 × 10^−2^ M AgNO_3_ water solution was added. The solution was gently stirred for 15 min at 30 °C, and then 500 μL of 5.0 × 10^−2^ M HAuCl_4_ water solution was added to the mixture, starting the pre-reduction step. At the selected pre-reduction time, 130 μL of 0.1 M ascorbic acid water solution was injected under strong stirring, followed by 80 μL of seed solution. After 30 s, the stirring was stopped and the mixture was left undisturbed for at least 4 h. The sample was finally centrifuged (9000 rpm, 20 min, 30 °C).

**Synthesis of AuNR@PEG-SH**

To 50 mL aqueous solution of CTAB-coated nanorods, 1 mL aqueous solution of PEG-SH (1 × 10^−4^ M) was added. CTAB, which was bound to the metal surface by electrostatic bonding, was easily displaced by the SH group, which formed a covalent bond with Au [45]. The sample was stirred vigorously overnight. It was then centrifuged and the pellet dispersed in water.

**Synthesis of AuNS@(Ir_1_)SiO_2_@TPSA and (Ir_1_)SiO_2_@TPSA**

Quaternary water/oil (W/O) microemulsion was obtained by mixing 7.2 mL of Triton X-100 (surfactant), 7.2 mL of n-hexanol (co-surfactant), 30 mL of cyclohexane (oil) and a water solution consisting of a mixture of 1.8 mL HAuCl_4_⋅3H_2_O (12.75 mM) water solution, 1.8 mL MES (36.5 mM) water solution and 0.6 mL NaBH_4_ (423 mM) water solution, in order to synthesize the gold nanoparticles. After 5 min, 0.1 mL of the corresponding metal complex solution, obtained by direct dissolution of 1 mg of **Ir_1_** in 0.1 mL of water, was added to the microemulsion, followed by 0.020 mL of APTES and 0.300 mL of TEOS. After 30 min, the silica polymerization reaction was completed by adding 0.160 mL of NH_4_OH (25%) water solution, which was then left stirring overnight. A protective charged shell was then added to the particles to provide colloidal stability in aqueous solution. A two-step addition of 0.030 mL of N-(3-triethoxysilyl) propylsuccinic anhydride after 24 and 48 h from the core formation ensured the long-term electrostatic stability of the colloid. Then, by the addition of isopropanol and water in a volume ratio 1:1:1, the microemulsion was broken. Further purification steps by ultrafiltration method (by using a crossflow device Sartorius Vivaflow^®^ 200 equipped with 100 KDa membranes) allowed the complete removal of all unreacted species. Gold–silica nanoparticles were finally dispersed in water and filtered by a 200 nm nylon membrane.

(**Ir_1_**)SiO_2_@TPSA was synthesized following the above procedure, but without the addition of **Ir_1_**.

**Synthesis of AuNR@SiO_2_@TPSA.** An amount of 10 μL of 0.2 M CTAB was mixed with 2 mL of an aqueous dispersion of AuNR@CTAB (2.5 × 10^−4^ M). After a few seconds, 20 μL of a 0.1 M NaOH aqueous solution was added under vigorous stirring, followed by three additions, each of 12 μL, of TEOS 20% *v*/*v* in methanol under mild stirring. After 14 h, the mixture was centrifuged in EtOH twice at 6000 rpm for 10 min [46]. Successively, a solution of 2 mL of EtOH containing 5 μL of TPSA was added, and gently stirred overnight. The mixture was finally centrifuged and dispersed in water.

**Synthesis of AuNR@(Ir_1_)SiO_2_@TPSA**. An amount of 6 mg of **Ir_1_** was dissolved in 2 mL of an aqueous dispersion of AuNR@CTAB (2.5 × 10^−4^ M). Henceforth the synthesis proceeds as in the previous case. After three purification steps, the washing waters, tested by spectroscopy, were free from iridium(III) complex.

**Synthesis of AuNR@SiO_2_(Ir_1_)SiO_2_@TPSA**. An amount of 10 μL of 0.2 M CTAB was added to 2 mL of an aqueous dispersion of AuNR@CTAB (2.5 × 10^−4^ M). After a few seconds, 20 μL of a 0.1 M NaOH aqueous solution was added under vigorous stirring, followed by TEOS 20% *v*/*v* (12 μL) in methanol under gentle stirring. An amount of 2 mL of an aqueous solution containing 6 mg of **Ir_1_** was then added, followed by two additions, each of 12 μL, of TEOS 20% *v*/*v* in methanol under gentle stirring. After 14 h, the mixture was centrifuged in EtOH twice at 6000 rpm for 10 min [46]. Successively, a solution of 2 mL of EtOH containing 5 μL of TPSA was added and gently stirred overnight. Finally, the mixture was centrifuged and dispersed in water.

**Figure 1:**
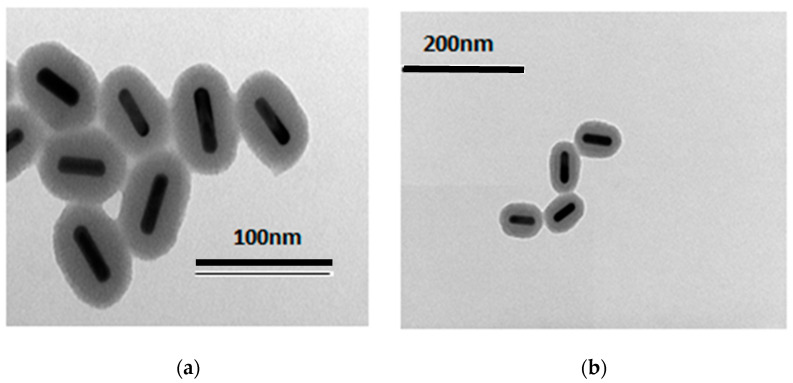
TEM images of AuNR@(**Ir_1_**)SiO_2_@TPSA (**a**) (total particle size 34 × 66 nm, short axis × long axis, respectively) and of AuNR@SiO_2_@TPSA (**b**) (total particle size 30 × 63 nm short axis × long axis, respectively).

**TEM images.** Samples **AuNR@(Ir_1_)SiO_2_@TPSA** and **AuNR@SiO_2_@TPSA** were observed by TEM (Figure 1). Although the followed synthetic procedures are identical, the coating thickness is different; in particular, the thickness of AuNR@(**Ir_1_**)SiO_2_@TPSA is greater than that of AuNR@SiO_2_@TPSA, i.e., 22 × 18 nm vs. 18 × 15 nm, short axis × long axis, respectively.

**Photophysical properties.** The photophysical characterization of **Ir_1_** has been published in various papers [14,42,47]. Here, we summarize the principal features. The absorption spectrum of **Ir_1_** (Figure S2) is dominated, in the UV range, by the intense spin-allowed ^1^π–π* ^1^LC transitions of aromatic ligands. In the 350–450 nm range, less-intense ^1^MLCT bands are visible. In addition, the direct spin-forbidden population of triplet excited states is responsible for the weak tails observed above 450 nm. (^3^MLCT and ^3^LC transitions). In fact, the high spin-orbit coupling of the iridium metal core allows the mixing of triplet states with the higher-lying ^1^MLCT levels. The presence of a high spin-orbit coupling increases the intersystem crossing efficiency from the singlet to the triplet excited states, with a final highly efficient spin-forbidden phosphorescence emission. **Ir_1_** in water solution (Figure S3) displays an intense luminescence band centered at 515 nm, due to the ^3^MLCT deactivation. As expected, the emission intensity is highly sensitive to the amount of oxygen. Time-dependent emission intensity of **Ir_1_** (Figure S4) has been fitted by a mono-exponential decay function. In Ar-equilibrated solutions, the complex shows a lifetime value of 2.182 μs that is reduced to 0.395 μs in the presence of oxygen. As expected, the emission quantum yield decreases from the value of 0.96 measured in the absence of oxygen, to 0.10 in an air-equilibrated solution.

**Figure 2:**
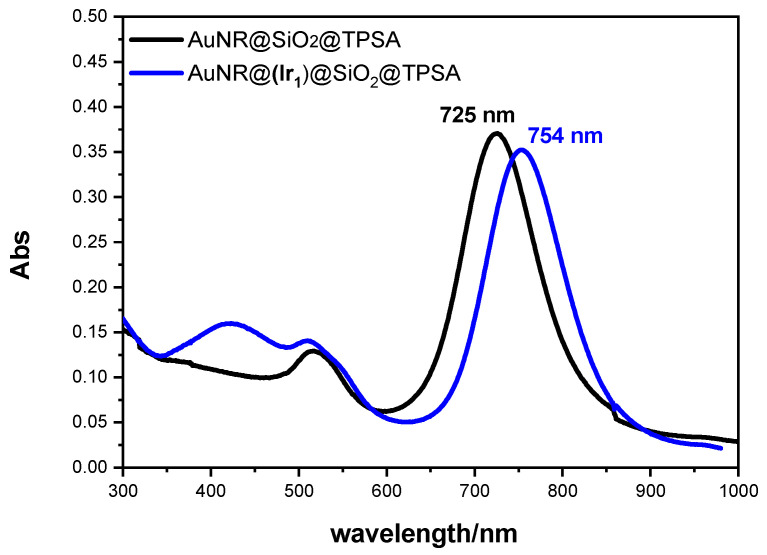
Extinction spectra of AuNR@(**Ir_1_**)SiO_2_@TPSA and of AuNR@SiO_2_@TPSA in water.

Figure 2 reports the extinction spectra of AuNR@(**Ir_1_**)SiO_2_@TPSA and AuNR@SiO_2_@TPSA in water solution. While the transverse plasmon band remains virtually unchanged, the longitudinal one appears red shifted from 725 to 754 nm in the NRs incorporating **Ir_1_**, compared with those without it. Furthermore, an absorption band of the complex at 420 nm is visible in the spectrum of AuNR@(**Ir_1_**)SiO_2_@TPSA (when compared with the spectrum in Figure S2).

**Figure 3:**
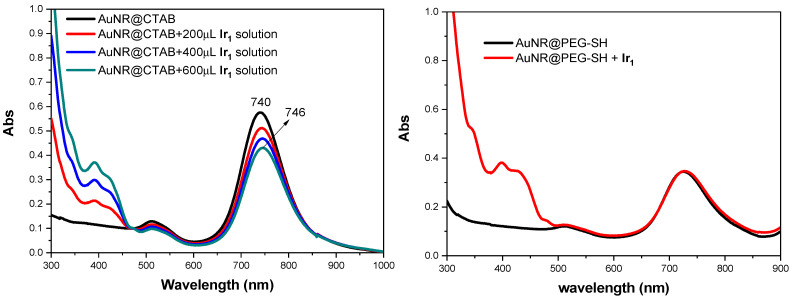
Spectroscopic result of the addition of **Ir_1_** to a solution of AuNR@CTAB (extinction spectra in water on the **left**) and to a solution of AuNR@PEG-SH (extinction spectra in ethanol on the **right**).

It is known that the longitudinal band position of AuNRs is significantly influenced by the refractive index of the dielectric in contact with the metal surface [46,48,49]. Because the coating agent in both samples is silica, which grows by modeling on the CTAB layer, the presence of **Ir_1_** in AuNR@(**Ir_1_**)SiO_2_@TPSA plays a relevant role on this layer. Considering that the iridium(III) complex has an ionic nature, it is plausible that the positively charged ion can partially displace the CTAB molecules from the gold, modifying the layer directly in contact with the metal. Furthermore, the CTAB micelles on the gold surface (which favor the ordered growth of silica [39]), are somehow disrupted and the silica, no longer confined inside the micelles, gives rise to greater shell thicknesses, as already observed for the AuNR@(**Ir_1_**)SiO_2_@TPSA sample with respect to AuNR@SiO_2_@TPSA (see above in Figure 1). To verify that **Ir_1_** can displace the CTAB layer causing a longitudinal band red shift, growing amounts of this complex were added to a water solution of AuNR@CTAB; the obtained results, reported in Figure 3 (on the left), show an increasing red shift, confirming this effect. **Ir_1_** can replace the CTAB layer because CTAB molecules are held onto metal surface by electrostatic interactions. If the AuNR coating was made by PEG-SH (which adheres to gold by a strong S-Au bond), **Ir_1_** cannot replace it, and consequently no red shift should be observed. To better confirm this hypothesis, **Ir_1_** was added to an alcoholic solution of AuNR@PEG-SH, and, in the relative extinction spectrum, no red shift is observed (Figure 3, on the right).

**Figure 4:**
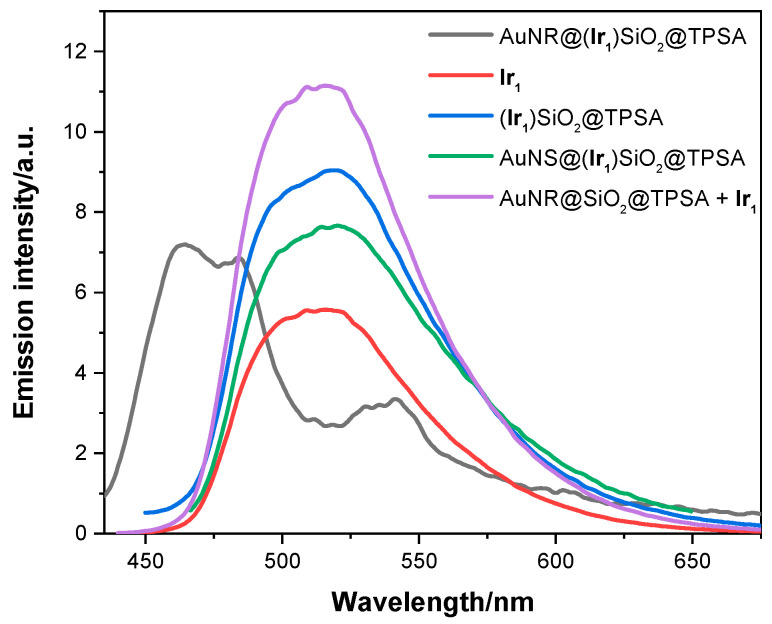
Emission spectra of AuNR@(**Ir_1_**)SiO_2_@TPSA, **Ir_1_**, (**Ir_1_**)SiO_2_@TPSA, AuNS@(**Ir_1_**)SiO_2_@TPSA and AuNR@SiO_2_@TPSA + **Ir_1_** in water obtained by exciting at 390 nm.

Then, the aqueous solutions of AuNR@SiO_2_@TPSA and AuNR@(**Ir_1_**)SiO_2_@TPSA were studied to verify their luminescence properties. While AuNR@SiO_2_@TPSA, as expected, does not show any emission, AuNR@(**Ir_1_**)SiO_2_@TPSA, which contains the fluorophore **Ir_1_**, shows a rather structured emission spectrum (Figure 4), with two bands in the blue and green range of the visible spectrum, each displaying two vibronic maxima, at 465 and 484 nm and at 515 and 542 nm, respectively.

Comparing this spectral profile with the emission of **Ir_1_** in solution reported in Figure 4, it is clearly noted that, while the band in the green range is superimposable to the emission of the complex (although slightly red shifted), the band in the blue range deserves a more in-depth study. To evaluate the contribution of each component of the AuNR@(**Ir_1_**)SiO_2_@TPSA nanoplatform, we compared its emission spectrum with that of a sample in which **Ir_1_** is embedded into silica nanoparticle (**Ir_1_**)SiO_2_@TPSA [14], in order to highlight the role of the plasmonic nanoparticle; subsequently, to evaluate the role of the plasmonic structure, we analyzed an identical system that had a spherical gold core, AuNS@(**Ir_1_**)SiO_2_@TPSA [14], instead of a rod-shaped one. Spectral profiles are compared in Figure 4, while lifetime measurements are reported in Table 1.

Considering the emission intensity decay of **Ir_1_** encapsulated in TPSA-coated silica spheres (i.e., (**Ir_1_**)SiO_2_@TPSA sample), we note that, compared with **Ir_1_** in aqueous solution, a longer lifetime appears, while the value of the short lifetime remains almost identical. The short lifetime (415 ns) can be attributed to the **Ir_1_** fixed on the nanoparticle surface (in contact with the solvent, and thus in an environment similar to **Ir_1_** in solution), while the longer one (2372 ns) is attributed to the decay of the iridium(III) complex embedded in the rigid silica shell. This is confirmed by the data obtained from the AuNS@(**Ir_1_**)SiO_2_@TPSA sample, which differs from (**Ir_1_**)SiO_2_@TPSA by virtue of the presence of the spherical gold core. In this case, we measured a short lifetime of 408 ns (attributable to the **Ir_1_** on the nanoparticle surface), and a long lifetime of 1210 ns (attributed to the **Ir_1_** into the silica matrix) that was reduced with respect to the value measured for (**Ir_1_**)SiO_2_@TPSA by an energy transfer process towards the gold plasmon, which peaked at 515 nm and therefore overlapped with the emission band of the fluorophore.

AuNR@(**Ir_1_**)SiO_2_@TPSA shows quite different photophysical properties compared with the previous samples; here, the emission spectrum features two main peaks, one located at 542 nm (similar to that shown by the previous samples at 515 nm, but now slightly red shifted), and a new band at 465 nm, absent in the previous samples. The time-resolved decays of the luminescence intensity were recorded at the maxima of the two spectral regions, i.e., at 465 and 542 nm, and the fitting procedure, using a tri-exponential function (with a chi-square of 1.28), gives the following three lifetime values: τ_1_ = 7 ns, τ_2_ = 83 ns, τ_3_ = 869 ns and τ_1_ = 18 ns, τ_2_ = 279 ns, τ_3_ = 1060 ns, respectively (see Table 1), with different values of amplitude of the components (α) depending on the emission peak.

To rationalize the properties of AuNR@(**Ir_1_**)SiO_2_@TPSA, it is useful to compare the photophysical features with those of AuNS@(**Ir_1_**)SiO_2_@TPSA, which differs from the previous sample because of the shape of the gold core and because of the synthetic procedure followed; in fact, in the case of gold nanospheres, the microemulsion method was employed [14], which uses gold nanospheres coated with MES on which TEOS polymerizes. In the case of gold nanorods, TEOS polymerizes on CTAB-coated rods. The microemulsion technique allows one to obtain isotropic nanoparticles homogeneous in shape and size, but it is not as versatile as the seed-mediated growth, thanks to which it is possible to obtain anisotropic nanoparticles of different shapes and sizes. In fact, though with this technique it is more difficult to obtain homogeneous samples, it is nevertheless widely used due to its versatility [45,50]. The microemulsion method allows one to obtain spherical nanoparticles the radius of which depends on the precursor ion concentration and surfactant to water ratio. The seed-mediated approach, indeed, allows one to obtain nanoparticles of different shapes and sizes. A key role in achieving the desired shape is played by the coating agent and the so-called shape directors (in our case CTAB as coating agent and silver nitrate as shape director). Furthermore, a bimodal reducing agent system consisting of the combination of salicylic acid and ascorbic acid allows for good control of the gold reduction at each stage of the nanorod formation, which improves the morphology control.

We propose that the high-energy and relatively high-intensity emission band at 465 nm of AuNR@(**Ir_1_**)SiO_2_@TPSA is ascribable to the **Ir_1_** directly in contact with the metal. The possibility of a direct contact of the fluorophore with the metal surface is due to the ionic nature of the Ir(III) complex that allows a partial substitution of CTAB molecules on the gold surface, while this is prevented in the analogous AuNS@(**Ir_1_**)SiO_2_@TPSA by the strong bond between MES and gold.

From a photophysical point of view, the interactions of fluorophores with metal surfaces show several effects, including increased quantum yields, higher photostability, longer FRET distances, and reduced lifetimes. These effects are called metal-enhanced fluorescence (MEF) [11,12,51]. A fluorophore in the excited state has the properties of an oscillating dipole. The excited fluorophore can induce oscillations of the electrons in the metal. The electric field created by the metal can interact with the excited fluorophore and alter its emission. In our case, however, we observe that the interaction between the metal surface of the nanoparticle with the fluorophore does not lead to an increase in the intensity of the typical **Ir_1_** emission band centered at 515 nm, but rather to the appearance of a new, rather structured band, at 465 nm, while the 515 nm band appears red shifted with a maximum at 542 nm.

It has been reported in many photophysical studies [52,53,54] that the luminescence of cyclometalated complexes of iridium(III) always derives from triplet levels, ^3^MLCT or ^3^LC in nature, which actually include a changeable amount of the corresponding singlets. We preliminarily propose that **Ir_1_** molecules in direct contact with the metal surface of a nanoparticle undergo a “demixing” of the LC and MLCT states, so that it is possible to observe the ^1^LC emission that we identify with the 465 nm band and that is responsible for the short lifetime, while the ^3^MLCT results are red shifted at 542 nm.

**Figure 5:**
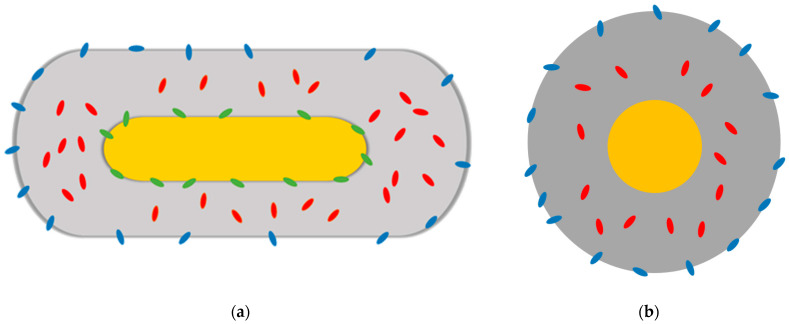
Graphical representation of the different location of **Ir_1_** in AuNR@(**Ir_1_**)SiO_2_@TPSA (**a**) and in AuNS@(**Ir_1_**)SiO_2_@TPSA (**b**). The green ellipses represent **Ir_1_** directly in contact with the gold surface; the red ellipses refer to a localization in the silica matrix; finally, the blue ellipses represent **Ir_1_** on the silica surface.

Based on this hypothesis, we can summarize the photophysical properties of AuNR@(**Ir_1_**)SiO_2_@TPSA and AuNS@(**Ir_1_**)SiO_2_@TPSA by attributing the two emission bands and the different measured lifetimes to a different localization of **Ir_1_** inside the gold nanoparticle. In particular, as shown in Figure 5, in the nanorods (a) and the nanospheres (b) the complexes present inside and on the surface of the siloxane matrix are responsible for the emission at 515 nm and the long and intermediate lifetimes; in the case of nanorods, where it is possible to hypothesize the presence of **Ir_1_** in contact with the gold surface, **Ir_1_** is responsible for the emission at 465 nm and the short lifetime, and of the red shifted band at 542 nm.

The lifetime values (τ_i_) and the relative amplitudes of the components (α_i_) can be interpreted based on this model. Indeed, in the case of AuNR@(**Ir_1_**)SiO_2_@TPSA, it is a single fluorophore (**Ir_1_**) that exists in different environments, and the values of α_i_ and τ_i_ can be used to determine the fractional contribution (*f*_i_) of each decay time to the steady-state intensity, calculated according to the following formula [11,51]:

$$f_{i\;}=\frac{\alpha_{i}\tau_{i}}{\sum_{j}{\alpha_{j}\tau }_{j}}$$

As discussed above, the lifetimes measured at 465 nm, mainly attributable to the ^1^LC levels, are shorter than those measured at 542 nm which originate from the decay of the ^3^MLCT state. Multi-exponential analysis shows that, at both wavelengths, there is a contribution from the different environments in which the **Ir_1_** chromophore is located inside the nanostructure. The value τ_1_ is attributed to the fraction of the Ir(III) complex in contact with the gold core, the time τ_2_ originates from the decay of the chromophore fraction on the nanoparticle surface, and, finally, the time value τ_3_ is attributed to the fraction dispersed in the siloxane matrix. As can be observed from the f_i_ values reported in Table 2, these contributions are different depending on the observation wavelength: at 465 nm the contribution of t_1_ is greater than that measured at 542 nm, and this confirms the attribution of the emission at 465 nm to the ^1^LC state of the **Ir_1_** fraction in direct contact with the gold core. At both wavelengths, the highest value of the fractional intensities is clearly that due to τ_2_, as the siloxane matrix is the one capable of hosting the largest amount of fluorophore molecules. The longest lifetime is the τ_2_ value, i.e., the one due to the **Ir_1_** fraction located in the siloxane matrix. Although this fraction is closer to the gold core than the one on the nanoparticle’s surface, and therefore more subject to energy transfer towards the plasmon, this quenching is compensated for by the greater rigidity of the environment compared with the **Ir_1_** fraction in direct contact with the aqueous environment in which the nanoparticles are dispersed.

**Figure 6:**
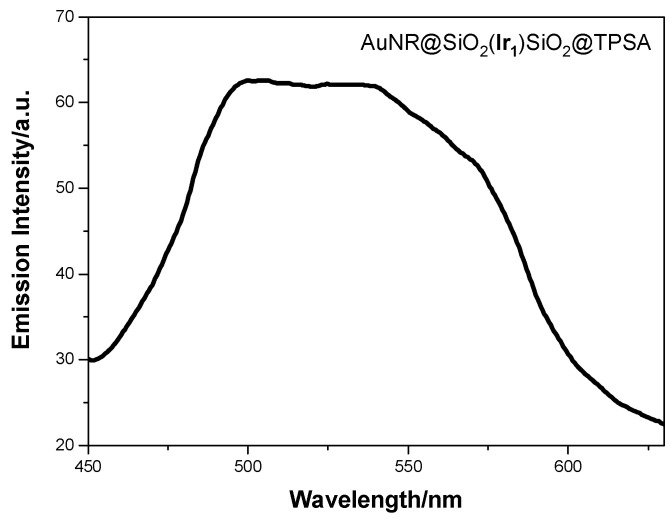
Emission spectra of AuNR@SiO_2_(**Ir_1_**)SiO_2_@TPSA obtained by exciting the iridium(III) complex at 390 nm.

The direct contact of the **Ir_1_** fluorophore with the metal surface determines the emission band at 465 nm. To better confirm this, we prepared AuNR@SiO_2_(**Ir_1_**)SiO_2_@TPSA, which differs from AuNR@(**Ir_1_**)SiO_2_@TPSA because fluorophore was added after the first TEOS addition (i.e., between the first and the second TEOS addition, see protocol “*Synthesis of AuNR@SiO_2_@TPSA*”): in this way **Ir_1_** was placed onto the first layer of silica shell, thus avoiding direct contact with the metal surface. In Figure 6, we report the emission spectrum of the sample AuNR@SiO_2_(**Ir_1_**)SiO_2_@TPSA. By comparing this spectrum with that obtained from AuNR@(**Ir_1_**)SiO_2_@TPSA we have safely shown the absence of the emission band at 465 nm due to the absence of a direct contact fluorophore-metal.

## 4. Conclusions

Fluorescence has always been a technique of great interest in various fields, due to its selectivity and sensitivity. In recent years, plasmonic nanomaterials have opened new avenues for the study and application of light–matter interaction processes. Nowadays, great efforts are being made to design, realize and investigate new nano-sized materials that combine the plasmonic properties of metal nanoparticles with the properties of luminescent molecules. The “talking” between the excited states of a molecule and the plasmonic resonator, the extent of which depends on the overlap between the emission band of the fluorophore and the plasmonic band of the nanoparticle, has been studied according to two main models, the FRET and the MEF, which, depending on the chromophore–nanoparticle distance and the shape of the latter, determines a quenching or an increase in the luminescence intensity. To the best of our knowledge, there are no studies that take into account the emitter specificity.

For many years our research group has been engaged in the synthesis and photophysical characterization of transition metal complexes [55] and in the synthesis of gold nanoparticles, in an effort to study the interplay between these two photoresponsive materials [56,57]. In this work we have synthesized nanosystems consisting of silica-coated gold nanorods incorporating our iridium(III) cyclometalated complex, in order to study the coupling effects of their optical properties.

Surprisingly, we observed a sort of splitting of the emission band of the metal complex, finally presenting an emission spectrum with two bands, one in the blue and the other one in the green-orange spectral range. Furthermore, we measured three values of the excited state lifetime, which we attributed to different localizations of the iridium(III) complex in the siloxane matrix at different distances from the surface of the metal core. To obtain a clearer picture of what we were observing, we synthesized analogous nano systems, from which it was possible to highlight the effect of different features. Based on what we observed, we proposed that the fraction of the iridium(III) complex in direct contact with the gold nanoparticle surface undergoes a “demixing” of the excited state, which for cyclometalated iridium complexes is generally considered a mixed LC + MLCT state.

Although in-depth theoretical and computational studies are still needed to clarify the mechanisms operating in these nanosystems, the typology of the fluorophore appears to be fundamental. As the extreme sensitivity of these new hybrid system metal cluster molecules is of considerable interest in biosensing, these results, although preliminary, constitute an important stimulus to continue research on this promising path.

## Figures and Tables

**Table 1:** Lifetimes measured in air-equilibrated water samples (reduced χ^2^ = 1.28). α represents the pre-exponential values.

Sample	Emission, λ_max_/nm	Lifetime, τ/ns (α/%)
**Ir_1_**	515	395
(**Ir_1_**)SiO_2_@TPSA	515	415 (56.4%); 2372 (43.6%)
AuNS@(**Ir_1_**)SiO_2_@TPSA	515	408 (31.9%); 1210 (68.1%)
AuNR@(**Ir_1_**)SiO_2_@TPSA	465	7 (15.4%); 83 (11.0%); 869 (63.6%)
	542	18 (2.7%); 279 (30.0%); 1060 (67.3%)

**Table 2:** Multiexponential analysis of the time-resolved emission intensity decays of AuNR@(**Ir_1_**)SiO_2_@TPSA.

Observation Wavelengths (nm)	Lifetimes (ns)			Pre-Exponential Factors			Fractional Intensities		
	τ_1_	τ_2_	τ_3_	α_1_	α_2_	α_3_	f_1_	f_2_	f_3_
465	7	83	869	0.254	0.11	0.636	0.00315	0.01620	0.98065
542	18	279	1060	0.027	0.300	0.673	0.00061	0.10494	0.89445

## Data Availability

Data is contained within the article or Supplementary Materials.

## Supplementary Materials

The following supporting information can be downloaded at https://www.mdpi.com/article/10.3390/nano14191543/s1. Figure S1: Molecular structure of Ir_1_; Figure S2: Absorption spectrum of Ir_1_ in water at room temperature (3.20 × 10^−5^ mol/L); Figure S3: Excitation spectrum (A) in degassed water (λ_em_ = 515 nm) and emission spectra of Ir_1_ at room temperature in an air-saturated environment (B) or in degassed (C) water (λ_ex_ = 260 nm); Figure S4: time-resolved emission decay of Ir_1_ in water (λ_ex_@379 nm, λ_monitored_ = λ_em_ = 510 nm); Figure S5: time-resolved emission decays of AuNR@(Ir_1_)@SiO2@TPSA in water (λ_ex_@379 nm, λ_monitored_ = λ_em_ = 465 nm (on the left), 540 nm (on the right)).
