# Supplementary material for: A “Talking” between Gold Nanoparticle and a Luminescent Iridium(III) Complex: A Study of the Effect Due to the Interaction between Plasmon Resonance and a Fluorophore
Source: Nanomaterials (Basel). 2024 Sep 24;14(19):1543. doi: 10.3390/nano14191543 (PMC11477608; doi:10.3390/nano14191543)
Supplement: Supplementary file 1 [file nanomaterials-14-01543-s001.zip › nanomaterials-3169022-supplementary.pdf]

A "talking" between gold nanoparticle and a luminescent Iridium(III) complex: a study of the effect due to the interaction between plasmon resonance and a fluorophore

Angela Candreva,[1,2] Loredana Ricciardi,[2] Elisabeta I. Szerb,[3] and Massimo La Deda [1,2]*

[1] Department of Chemistry and Chemical Technologies, University of Calabria, I-87036 Rende (CS), Italy

[2] CNR-NANOTEC Institute of Nanotechnology, National Research Council, I-87036 Rende (CS), Italy

[3] Coriolan Dragulescu Institute of Chemistry, Romanian Academy, 24, Mihai Viteazu Bvd., 300223-Timisoara, Romania

* Correspondence: massimo.ladeda@unical.it

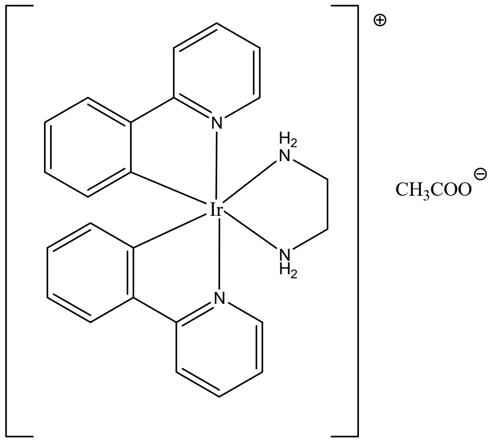

Figure S1. Molecular structure of $\mathbf{Ir_1}$.

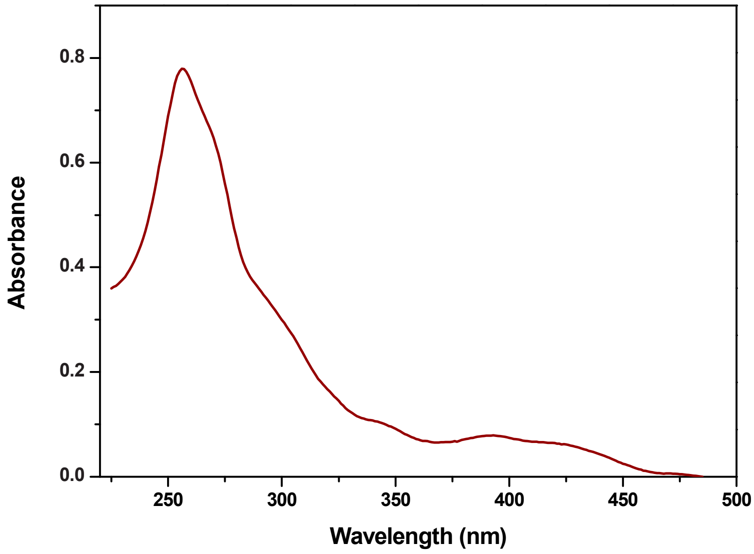

Figure S2. Absorption spectrum of $\mathbf{Ir_1}$ in water at room temperature (3.20×10^{-5} mol/L).

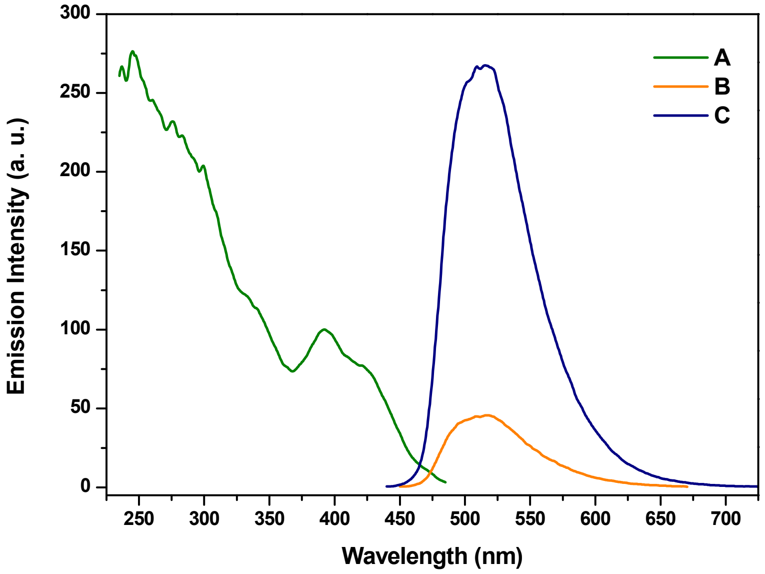

Figure S3. Excitation spectrum (**A**) in degassed water (λ_{em}= 515 nm) and emission spectra of **Ir$_1$** at room temperature in air-saturated (**B**) or in degassed (**C**) water (λ_{ex}= 260 nm).

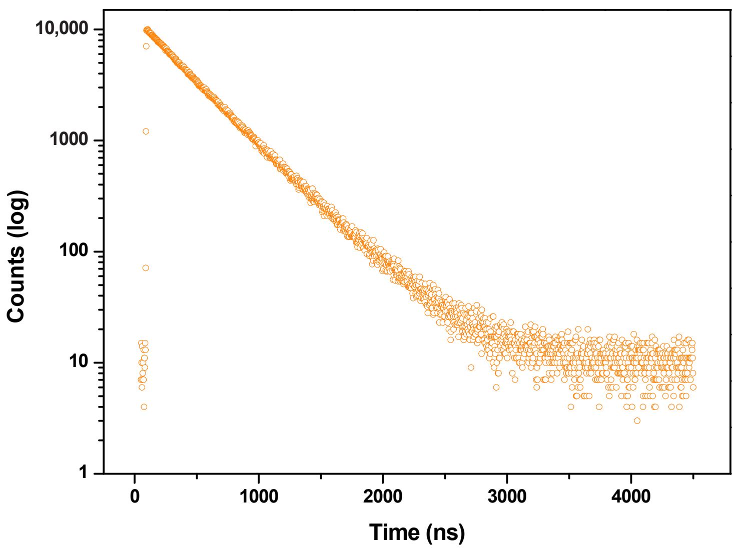

Figure S4 Time-resolved emission decay of **Ir$_1$** in water (λ_{ex}= 379 nm, $\lambda_{monitored}$= λ_{em}= 510 nm).

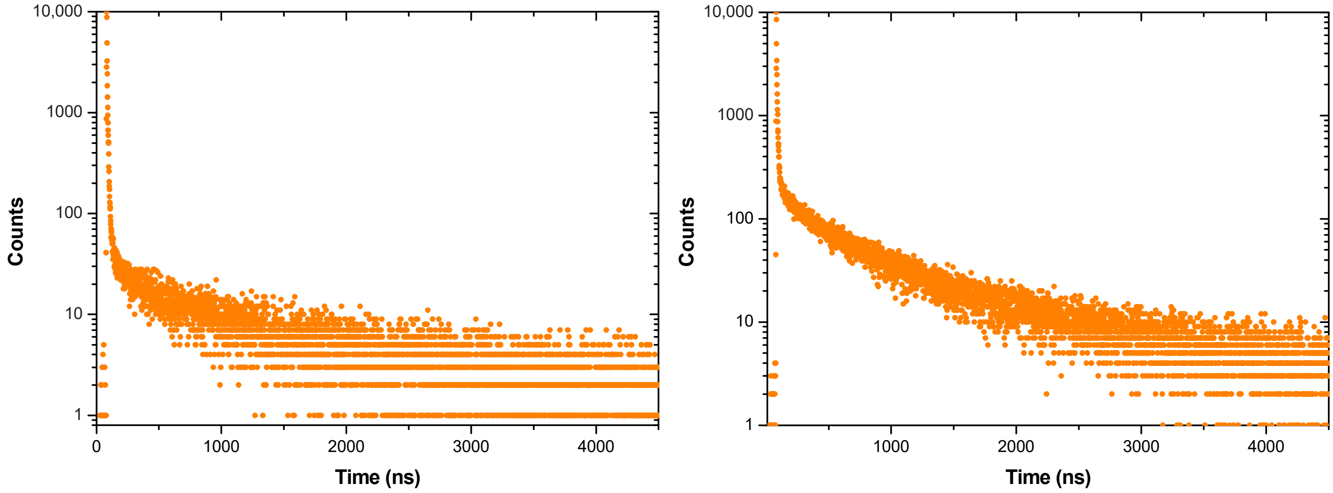

Figure S5. Time-resolved emission decays of AuNR@(**Ir$_1$**)@SiO_2@TPSA in water (λ_{ex}= 379 nm, $\lambda_{monitored}$= λ_{em}= 465 nm (on the left), 540 nm (on the right)).
